# Book Reviews

**Published:** 1893-08

**Authors:** 


LECTURES ON MENTAL DISEASES, DESIGNED FOR MEDICAL STUDENTS AND GENERAL PRACTITIONERS, by Henry Putnam Stearns, A. M., M. D., physician Superintendent of the Bradford Retreat, etc., published by P. Blakiston, Son & Co., Philadelphia, 1893.

Dr. Stearns is modest when he announces in his title, for students and general medical practitioners, for the work deserves perusal from the specialist as well. His style is good and his subject well handled. The book is gotten up in good form.

The appendix is of special value, as giving an epitome of the codes medico-legal of the different States, and will prove of much service as a ready reference.

